# Annotations

**Published:** 1899-07-22

**Authors:** 


### Annotations.

### University Extension Lectures for Medical Men.

The Victoria University, being a modern and somewhat go-ahead institution, has arranged to carry post-graduate teaching to the doctors instead of waiting for them to come up to the great centres of learning. In other words, it has arranged courses of "extension lectures" to medical men on medical topics in certain local centres. The Chester Medical Society recently organised a series of lectures under this scheme to be given at the Chester General Infirmary by Professor Delépine, and these, we understand, have been well attended by the medical men in the neighbourhood. A somewhat similar plan was, we believe, in operation in certain parts of America some time back, but not in direct connection with a university. The sanction of some permanently established public body such as a university seems, however, to be essential to the success—and we may add to the professional propriety—of any such scheme. It would be a terrible prospect if volunteer medical lecturers—rising specialists of various degrees—were to take to "starring the provinces," and casting their bread upon the waters in the form of popular lectures suited to the taste of the "busy general practitioner." But even as part of an "extension" scheme we do not think that this plan is entirely without drawbacks. It seems likely, we fear, to foster the idea that local men must always sit at the feet of the great authorities. As a means of awaking interest such courses of lectures may do much good, and we have no doubt that those we have referred to have been very useful; but we know that nothing so much damps the ardour of those who would do good work, even in small towns, than the feeling that everything of importance is referred over their heads to the great teaching centres. This is an evil, and we cannot but see that the "star system," unless dealt with in the most careful possible manner, is likely to be as disastrous to local talent as it has been in another profession, and without the compensating advantages.

### The Aerial Convection of Small-Pox.

Writing to the *Times* a week or two ago, Professor Haffkine incidentally raised a point which, we think, will have to be very carefully considered before long. We have already stated several times that, in our opinion, the time has come when the whole question of the aërial convection of small-pox infection requires re-investigating. The statistics given in the classical investigations made by Dr. Power and Dr. Barry are, of course, unimpeachable; but a good deal of knowledge has of late years been gathered about flies as carriers of disease, and if it should turn out that flies are the active agents in spreading small-pox, it clearly would in many cases be much cheaper to intercept the out-going fly than to conform to recent Local Government Board regulations in regard to the distance which should intervene between a small-pox hospital and surrounding houses. Mr. Haffkine said that small-pox is "a purely contagious disease," and is spread "by contact with the patient or with objects that had come in direct contact with him, with his clothing, bedding, or with flies and other insects transferring the contagion from man to man, &c. It is certain that there exists not a single demonstration free from the gravest objections to show that small-pox can be contracted from food, or the water supply, or from merely breathing the air in a small-pox area, or from any 'emanation' from a small-pox patient, and so on." Years ago it was suggested by the Hospitals Commission that the air passing out from the wards of a small-pox hospital should be made to pass through a furnace, with the object of destroying any living infection which it might contain, a process, however, which, if only from economical considerations, might be attended with considerable difficulty. If, however, it should turn out that the "contagium" or infectious particle is always carried by insects or by dust, that is, by substance of tangible size, something less than complete destruction might be enough, and where the placing of small-pox hospitals in the midst of inhabited areas seems unavoidable, it might be sufficient to filter the outgoing air in the same manner as the ingoing air is treated in hospitals ventilated on the plenum system. The whole subject of aërial convection is in a double sense "in the air," and requires to be investigated afresh.

### The Rating of Charitable Institutions.

A curious case has arisen in the neighbourhood of Glasgow which forcibly draws attention to the necessity of coming to some general conclusion as to the rating of charitable institutions. The Orphan Homes of Scotland, usually called, from the name of their founder, "Quarrier's Homes," are situated at Bridge of Weir, in the parish of Kilmalcolm. They contain about a thousand children. Hitherto these homes have been exempted from rates by the good-will of the parish authorities, but this year these decided that the Homes must pay the assessment like all other houses. It seemed to them that it was a pity not to add the large sum to be thus obtained to the resources of the parish. Mr. Quarrier felt aggrieved by this action. He paid the rates, however, but then demanded that the children in the Homes should be received at the schools in the parish for their education. If the institution was to be rated, the children in it had the right to free education at the expense of the parish, as all ratepayers' children, and indeed all children, had. The School Board was, of course, quite unprepared to receive into their schools, already well filled, rather more than a thousand new children. Moreover, they argued that these children did not belong to the parish, and therefore had no claim on it for education. They are drawn from every part of Scotland, and should, in the opinion of Kilmalcolm, be educated at the expense of the parishes to which they belonged, if education were not to be given them as a part of the charity that maintained them. Thus a very pretty quarrel ensued. Partisans on either side are keen, and it is felt that according to the decision come to by the parties interested—or by the law if they should fail to come to any terms—an important precedent will be established. Those who take Mr. Quarrier's side argue that if the children come from all parts of the country, so do the funds that maintain them. Probably Mr. Quarrier is right in arguing that if he pays rates he has a right to profit by all rate-provided institutions; but whether or no, it will be a matter for regret if he and the parish cannot come to some amicable arrangement.

